# Combination efficacy of pertuzumab and trastuzumab for trastuzumab emtansine-resistant cells exhibiting attenuated lysosomal trafficking or efflux pumps upregulation

**DOI:** 10.1007/s00280-020-04138-5

**Published:** 2020-09-30

**Authors:** Yoriko Yamashita-Kashima, Sei Shu, Masahiro Osada, Takaaki Fujimura, Shigeki Yoshiura, Naoki Harada, Yasushi Yoshimura

**Affiliations:** Product Research Department, Chugai Pharmaceutical Co., Ltd., 200 Kajiwara, Kamakura, Kanagawa 247-8530 Japan

**Keywords:** Pertuzumab, Trastuzumab, Trastuzumab emtansine, T-DM1 resistance, HER2

## Abstract

**Purpose:**

Trastuzumab emtansine (T-DM1) is the standard treatment in the current second-line therapy of human epidermal growth factor receptor 2 (HER2)-positive metastatic breast cancer. However, a useful therapy after T-DM1 resistance has not been established. In this study, we established two different HER2-positive T-DM1-resistant cancer cells and evaluated the antitumor effect of trastuzumab in combination with pertuzumab (TRAS + PER).

**Methods:**

Single-cell-cloned OE19 and BT-474 cells were cultured with increasing concentrations of T-DM1 to generate T-DM1-resistant OE19bTDR and BT-474bTDR cells, respectively. HER2 expression was assessed by immunohistochemistry. Multidrug resistance proteins (MDR1 and MRP1) were evaluated by real-time polymerase chain reaction and western blotting. Intracellular trafficking of T-DM1 was examined by flow cytometry and immunofluorescence staining. Efficacy of TRAS + PER was evaluated by cell proliferation assay, HER3 and AKT phosphorylation, caspase 3/7 activity, and antitumor activity.

**Results:**

HER2 expression of both resistant cells was equivalent to that of the parent cells. Overexpression of MDR1 and MRP1 was observed and affected the T-DM1 sensitivity in the OE19bTDR cells. Abnormal localization of T-DM1 into the lysosomes was observed in the BT-474bTDR cells. In BT-474bTDR cells, TRAS + PER inhibited the phosphorylation of AKT involved in HER2–HER3 signaling, and apoptosis induction and cell proliferation inhibition were significantly higher with TRAS + PER than with the individual drugs. TRAS + PER significantly suppressed tumor growth in the OE19bTDR xenograft model compared with each single agent.

**Conclusions:**

The results suggest that the TRAS + PER combination may be effective in T-DM1-resistant cancer cells where HER2 overexpression is maintained.

**Electronic supplementary material:**

The online version of this article (10.1007/s00280-020-04138-5) contains supplementary material, which is available to authorized users.

## Introduction

Trastuzumab emtansine (T-DM1) is composed of the human epidermal growth factor receptor 2 (HER2)-targeted humanized antibody trastuzumab (TRAS) and DM1, a maytansinoid derivative, linked with a non-reducible thioether linker, *N*-succinimidyl-4-(*N*-maleimidomethyl) cyclohexane-1-carboxylate (SMCC, designated MCC after conjugation). Upon binding to HER2 receptor, T-DM1 undergoes receptor-mediated internalization and subsequent lysosomal degradation, resulting in intracellular release of DM1-containing cytotoxic catabolites. Binding of DM1 to tubulin disrupts microtubule networks in cells, which results in cell cycle arrest and apoptotic cell death. In addition, T-DM1 has been reported to retain the mechanisms of action (MOAs) of TRAS [1–3].

TRAS is an anti-HER2 antibody that binds to domain IV of HER2; its antitumor MOAs include inhibition of ligand-independent HER2–HER3 signaling in HER2-amplified cells, antibody-dependent cellular cytotoxicity (ADCC), and inhibition of shedding of the extracellular domain of HER2 [4, 5]. Pertuzumab (PER), another anti-HER2 antibody, binds to domain II of HER2 in HER2-positive breast cancer cells; its MOAs include inhibition of ligand-dependent HER2–HER3 signaling by blocking heterodimerization of HER2 with other HER family members (including epidermal growth factor receptor [EGFR], HER3, and HER4) and ADCC [6, 7]. TRAS and PER inhibit HER2 signals through different mechanisms, thereby producing a synergistic effect [7–9].

Treatment guidelines, such as those from the American Society of Clinical Oncology [10], National Comprehensive Cancer Network [11], and Japanese Breast Cancer Society [12], recommend that in patients with HER2-positive metastatic breast cancer, the standard first-line therapy should include the TRAS + PER + docetaxel combination and second-line therapy should include T-DM1. This recommendation is based on results showing significant improvement in progression-free survival and/or overall survival in the phase 3 CLEOPATRA (TRAS + PER + docetaxel vs TRAS + docetaxel) [13, 14] and EMILIA (T-DM1 vs lapatinib + capecitabine [CAPE]) [15] studies.

As third-line therapy, the continuous use of a HER-targeting drug, such as TRAS or lapatinib, with chemotherapy has been reported to prolong progression-free survival in patients [16, 17]; however, data on the selection of anti-HER2 drugs in patients with T-DM1 resistance are lacking and require clarification. Recently, several possible mechanisms of T-DM1 resistance have been reported: (1) reduced binding of T-DM1 to HER2 by HER2 downregulation [18] or by mucin 4 (MUC4) [19]; (2) attenuation of AKT signal inhibition caused by phosphatase and tensin homolog (PTEN) loss [20]; (3) signaling via the EGFR [21]; (4) overexpression of adenosine triphosphate (ATP)-binding cassette (ABC) transporters that are involved in multidrug resistance [18, 20, 22]; (5) downregulation of the lysosomal transporter solute carrier family 46 member 2 (SLC46A2) [20, 23], lysosomal metabolic disorders [24, 25], or abnormal lysosomal trafficking [26]; or (6) altered microtubule dynamics through a mutation in tubulin or altered activation of mitotic regulators [27, 28]. Except for (1)–(3), these resistance mechanisms are specific to T-DM1 and not to TRAS or PER. Therefore, when HER2 expression and downstream signaling are normal and the HER2-binding site is not masked, a possible modality is to re-administer TRAS + PER for complete blocking of HER2 signaling. Therefore, the objective of the current study was to evaluate the antitumor effect of the TRAS + PER combination in HER2-positive T-DM1-resistant cell lines, thereby demonstrating the feasibility of re-administration of TRAS + PER after T-DM1 resistance has occurred in HER2-positive metastatic breast cancer.

## Materials and methods

### Test agents

T-DM1, TRAS, and PER were provided by Chugai Pharmaceutical Co., Ltd. Human immunoglobulin G (HuIgG) was purchased from MP Biomedicals, LLC. TRAS and HuIgG were dissolved in distilled water. All agents were diluted with saline for in vivo experiments and with culture medium for in vitro experiments. CAPE, obtained from Chugai Pharmaceutical Co., Ltd., was suspended in 40 mmol/L citrate buffer (pH 6.0) containing 5% gum Arabic as the vehicle. DM1-CH_3_ was purchased from Toronto Research Chemicals.

### Cell lines and culture conditions

HER2-positive human gastroesophageal junction cancer cell line OE19 was obtained from the European Collection of Authenticated Cell Cultures (catalog no. 96071721) and maintained in RPMI-1640 (Sigma-Aldrich catalog no. R8758), supplemented with 10% fetal bovine serum (Bovogen Biologicals catalog no. SFBS-C; Sigma-Aldrich catalog no. 172012; or Nichirei Biosciences catalog no. 174012), 1 mM sodium pyruvate, 10 mM HEPES, and 0.45% d-glucose. HER2-positive human breast cancer cell line BT-474 was obtained from the American Type Culture Collection (catalog no. HTB-20) and maintained in RPMI-1640 supplemented with 10% fetal bovine serum, 1 mM sodium pyruvate, 10 mM HEPES, 0.45% d-glucose, and 10 μg/mL bovine insulin. Cell lines were cultured at 37 °C under 5% CO_2_.

### Establishment of resistant cell lines

Single-cell cloning was performed on the OE19 and BT-474 cell lines to produce the OE19b and BT-474b cell lines, respectively. These were cultured with stepwise increase in concentrations of T-DM1 from 0.03 to 1.89 μg/mL and from 0.11 to 3.58 μg/mL for approximately 8 months to produce the T-DM1-resistant cell lines OE19bTDR and BT-474bTDR, respectively. OE19bTDR was maintained in culture medium with 1.89 μg/mL T-DM1, whereas BT-474bTDR was maintained in culture medium without T-DM1.

### In vitro antiproliferation assay

Cells seeded on 96-well plates at 1 × 10^4^ cells/well and precultured for 24 h were treated with T-DM1 or DM1-CH_3_ and incubated for 3 or 4 days. For the antiproliferation assay of T-DM1 in the presence of multidrug resistance protein 1 (MDR1) or multidrug resistance-associated protein 1 (MRP1) inhibitor, verapamil or MK-571 was added simultaneously with T-DM1. The cell number was analyzed with the blue fluorescent Hoechst 33258 nucleic acid stain as previously described [29]. For the antiproliferation assay of T-DM1 after knockdown of MDR1 or MRP1, the proliferation was analyzed with Cell Proliferation ELISA, BrdU (colorimetric) (Roche Diagnostics). For the antiproliferation assay of TRAS + PER, cells were treated with 40 μg/mL of HuIgG, TRAS, and/or PER, and with 20 ng/mL of heregulin β (HRGβ) 24 h later, and then cultured for 6 days. Proliferation was analyzed with Cell Counting Kit-8 (Dojindo Laboratories). The percentage of cell proliferation was calculated as follows: % proliferation = (measured value of treatment well − measured value of precultured well)/(measured value of non-treated well − measured value of precultured well) × 100.

To estimate the half maximal inhibitory concentration (IC_50_), the logarithmic-translated value of the drug concentration (*x* axis) and percentage of proliferation (*y* axis) were plotted and the two points across the IC_50_ value were fitted to a straight line. IC_50_ values were then estimated using the fitted line.

### HER2 protein expression (immunohistochemistry)

Cells were suspended and solidified in iPGell (GenoStaff). These were fixed with 10% neutral buffered formalin for 24 h and embedded in paraffin. HER2 protein expression was examined by immunohistochemistry (IHC) using HercepTest (Dako) at SRL Medisearch. HER2 scoring was determined by SRL Medisearch in accordance with the guidelines for gastric or breast cancer.

### Exome sequencing

Genomic DNA samples were extracted by a NucleoSpin Tissue Kit (Takara Bio). Next-generation sequencing was performed at Takara Bio. Sequencing library construction for human exome sequencing was done using Sure SelectXT Reagent Kit and Sure Select XT Human All Exon Kit V6 (Agilent Technologies). Sequencing was done using NovaSeq 6000 (Illumina).

### MDR1 and MRP1 mRNA expression

The levels of messenger RNA (mRNA) expression of MDR1/ATP-binding cassette subfamily B member 1 (ABCB1) and MRP1/ATP-binding cassette subfamily C member 1 (ABCC1) were determined using LightCycler 480 (Roche Diagnostics). Total RNA was extracted using RNeasy Mini Kit (Qiagen) and reverse transcribed using High-Capacity RNA-to-cDNA Kit (Thermo Fisher Scientific) and TaqMan probe/primer sets (ABCB1, Hs00184500_m1; ABCC1, Hs01561502_m1; Applied Biosystems).

### Western blotting

Whole cells were lysed in a cell lysis buffer (Cell Signaling Technology), containing a protease inhibitor cocktail (Sigma-Aldrich) and phosphatase inhibitor cocktail (Nacalai Tesque), and these lysates were fractionated on sodium dodecyl sulfate–polyacrylamide gel electrophoresis and transferred to polyvinylidene fluoride membranes using the iBlot 2 Dry Blotting System (Thermo Fisher Scientific) or were used for the capillary electrophoresis-based protein analysis system Sally Sue (ProteinSimple).

For the analysis of HER2–HER3 signal inhibition, cells were treated with HuIgG (as a control), TRAS (40 μg/mL), PER (40 μg/mL), or both for 3.5 h in serum-free medium and stimulated with 100 ng/mL of HRGβ for 5 min. Thereafter, cells were lysed as described above.

Primary antibodies against MDR1, HER2, pHER2, HER3, pHER3, AKT, PTEN, cell-division cycle protein 20 (CDC20), Aurora A, Aurora B, MAD2L1, cyclin B1, pAKT, β-actin (Cell Signaling Technology), MRP1, solute carrier family 46 member 3 (SLC46A3), BubR1, and cyclin-dependent kinase 1 (CDK1) (Abcam) were used.

### MDR efflux assay

Cells seeded on 96-well plates at 4 × 10^4^ cells/well and precultured for 24 h were treated with 2, 4, or 8 μM of verapamil or 5, 10, or 25 μM of MK-571, and incubated for 2.5 h. MDR pump efflux activity was then detected using an MDR Assay Kit (Abcam).

### Knockdown of MDR1 or MRP1

Cells seeded on six-well plates at 4 × 10^5^ cells/well and precultured for 24 h were transfected with ON-TARGET*plus* Human ABCB1 (5243) small interfering RNA (siRNA)-SMARTpool, ON-TARGET*plus* Human ABCC1 (4363) siRNA-SMARTpool, or ON-TARGET*plus* Non-targeting siRNA #4 (Dharmacon) using Lipofectamine RNAiMAX (Thermo Fisher Scientific). Following incubation and re-transfection, the cells were used for the antiproliferation assay of T-DM1.

### Assessment of mitotic spindle formation

Cells seeded on eight-well chamber slides at 3 × 10^4^ cells/well and precultured for 48 h were treated with T-DM1 and incubated for 48 h. The cells were washed and fixed with 4% paraformaldehyde for 10 min at room temperature (RT). The cells were then washed and permeabilized with 0.2% Triton X-100/phosphate buffered saline (PBS) for 15 min at RT. Thereafter, the cells were washed and blocked with blocking buffer (3% bovine serum albumin in PBS) for 1 h at RT. Primary antibody (anti-alpha tubulin antibody [Abcam]) and secondary antibody (anti-rabbit IgG [H + L], F[ab’]_2_ fragment [Alexa Fluor 555 Conjugate] [Cell Signaling Technology]) were added and incubated for 1 h at RT, respectively. After removing from the chamber, one drop of ProLong Gold Antifade Mountant with DAPI (Thermo Fisher Scientific) was added and a cover glass was placed. Fluorescence microscopy (Nikon, C1Si Confocal Microscope) at 60 × magnification was used to visualize the cells.

### Detection of T-DM1 on the cell surface

Cells seeded on six-well plates at 1 × 10^6^ cells and precultured were treated with 5 μg/mL of T-DM1 or HuIgG and incubated at 4 °C for 1 h. After changing the medium, cells were incubated at 37 °C for 0, 24, 48, or 72 h. The cells were collected and treated with PE Mouse Anti-Human IgG (BD Biosciences) and then incubated at 4 °C for 40 min. The samples were analyzed on the flow cytometer using FlowJo 10.4.1 (BD Biosciences).

### Assessment of intracellular T-DM1

Cells were seeded on 24-well plates with collagen1-coated cover glasses at 2.5 × 10^5^ cells and precultured. The cells were treated with Alexa-488-labeled T-DM1 or IgG (labeled using Zenon Alexa Fluor 488 Human IgG Labeling Kit [Thermo Fisher Scientific]) and incubated for 5 or 24 h. After washes, the cells were fixed with 4% paraformaldehyde for 15 min at 37 °C. The mountant used was ProLong Diamond Antifade Mountant (Thermo Fisher Scientific). Fluorescence microscopy at 60 × magnification was used to visualize the cells.

### Assessment of the localization of T-DM1 to lysosome

For the detection of T-DM1 transported to the lysosome, cells were seeded on 96-well black polystyrene microplate clear flat bottom (Corning) and precultured. T-DM1 or HuIgG was labeled with pHAb Amine Reactive Dyes (Promega). Cells were treated with T-DM1 or HuIgG-pHAb for 1 h at 4 °C and for 24 h at 37 °C. After incubation, the medium was exchanged with PBS and cells were visualized with fluorescence microscopy.

### Apoptosis assay

Cells were seeded on 96-well white-wall plates at 1 × 10^4^ cells/well and precultured for 24 h. The medium was changed to serum-free medium and cells were additionally precultured for 24 h. The cells were treated with 40 μg/mL of HuIgG, TRAS, and/or PER, and half an hour later, with 40 ng/mL of HRGβ, and then incubated for 24 h. Caspase 3/7 activity was assessed using the Caspase-Glo 3/7 Assay (Promega).

### Xenograft model

All animal experiments were performed in accordance with the Guidelines for the Care and Use of Laboratory Animals at Chugai Pharmaceutical Co., Ltd., and all animal procedures were reviewed and approved by the Institutional Animal Care and Use Committee at Chugai Pharmaceutical Co., Ltd. (approval number: 15-362). Five to seven-week-old male BALB/c-nu/nu mice (CAnN.Cg-*Foxn1*^*nu*^/CrlCrlj, purchased from Charles River Laboratories Japan) were inoculated subcutaneously in the right flank with 5 × 10^6^ cells/mouse with either OE19b or OE19bTDR cells. Several weeks after the inoculation, mice were randomized to the control group or treatment groups. T-DM1 (10 mg/kg) or saline was administered intravenously once every 3 weeks. TRAS (40 or 80 mg/kg) and/or PER (40 or 80 mg/kg), or HuIgG was administered intraperitoneally once a week for 3 weeks. CAPE (359 mg/kg) or vehicle was administered orally for the initial 14 days. Tumor volume and body weight were measured twice a week. Tumor volume was calculated as described previously [8].

### Statistical analyses

The Dunnett test was used to compare the means from multiple experimental groups with those of the control group. The Student’s *t* test was used to compare means of two different experimental groups. The Tukey–Kramer’s test was used for pairwise comparison of means between multiple groups. The Wilcoxon test was used to compare in vivo samples, and for multiple comparisons, hierarchical testing and Bonferroni correction were applied. *P* values < 0.05 or < 0.025 with Bonferroni correction were considered statistically significant, and the analysis was performed using JMP 11.2.1 (SAS Institute Japan Ltd.).

## Results

### Establishment and characterization of T-DM1-resistant cell lines

**Fig. 1:**
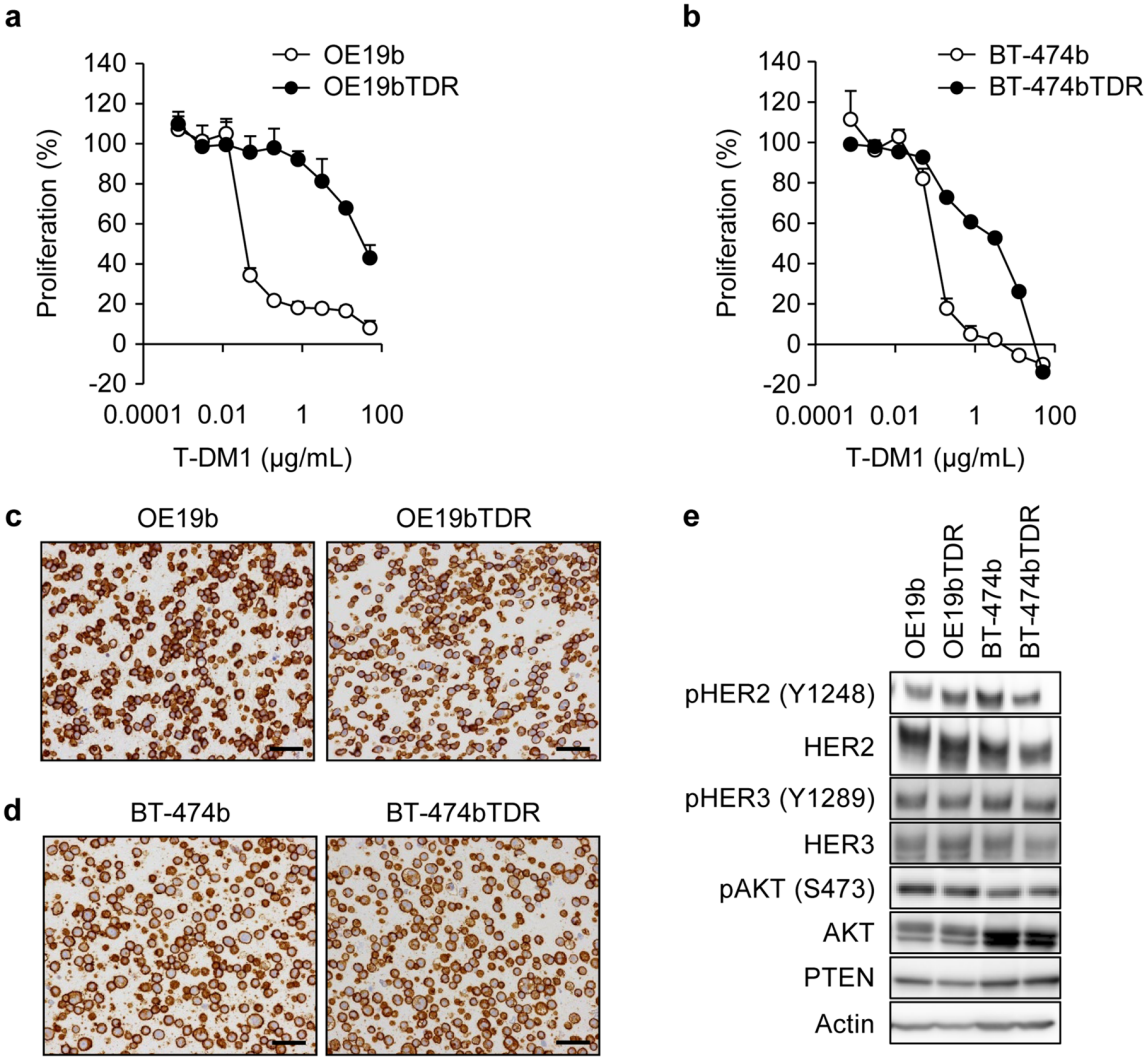
Characterization of the two T-DM1-resistant cell lines. Cell growth inhibition by T-DM1 was examined 4 days after treatment in the OE19b, OE19bTDR, **a** BT-474b, and BT-474bTDR cells. **b** Data points are mean + SD (*n* = 3). HER2 expression in the T-DM1-resistant cell lines was detected by immunohistochemistry. Black line represents 50 μm (**c**, **d**). HER2–HER3-AKT signaling was detected by western blotting (**e**)

Single-cell cloning was performed on the OE19 and BT-474 cell lines and the OE19b and BT-474b cell lines were established, respectively. The proliferation rate, HER2 expression, and sensitivity to T-DM1 of these cell lines were confirmed to be comparable with those of OE19 or BT-474. Furthermore, these were cultured in increasing concentrations of T-DM1 to produce the resistant cell lines OE19bTDR and BT-474bTDR, respectively. To characterize the two established T-DM1-resistant cell lines, sensitivity to T-DM1 and HER2 expression was assessed. The IC_50_ value of T-DM1 was approximately 900-fold in the OE19bTDR cells (34 μg/mL) than in the OE19b cells (0.036 μg/mL). The corresponding IC_50_ value of T-DM1 was approximately 40-fold in the BT-474bTDR cells (3.6 μg/mL) than in the BT-474b cells (0.098 μg/mL) (Fig. 1a, b). A positive HER2 expression represented by complete circumferential and strong membrane staining of more than 10% of cells (IHC score 3 +) was observed in both the resistant cell lines (Fig. 1c, d) (consistent with findings in Ref. [20, 21] for BT-474). HER2 downstream signaling in OE19bTDR and BT-474bTDR was also equivalent to that in the parental cells (Fig. 1e). We also assessed *ERBB2*, *ERBB3*, *PIK3CA*, *PIK3R1*, *PTEN*, *AKT1*, *PDK1* mutations, but there were no differences in functional mutations between parental and resistant cells (data not shown).

### Resistance to DM1-CH_3_ and expression of multidrug resistance proteins

**Fig. 2:**
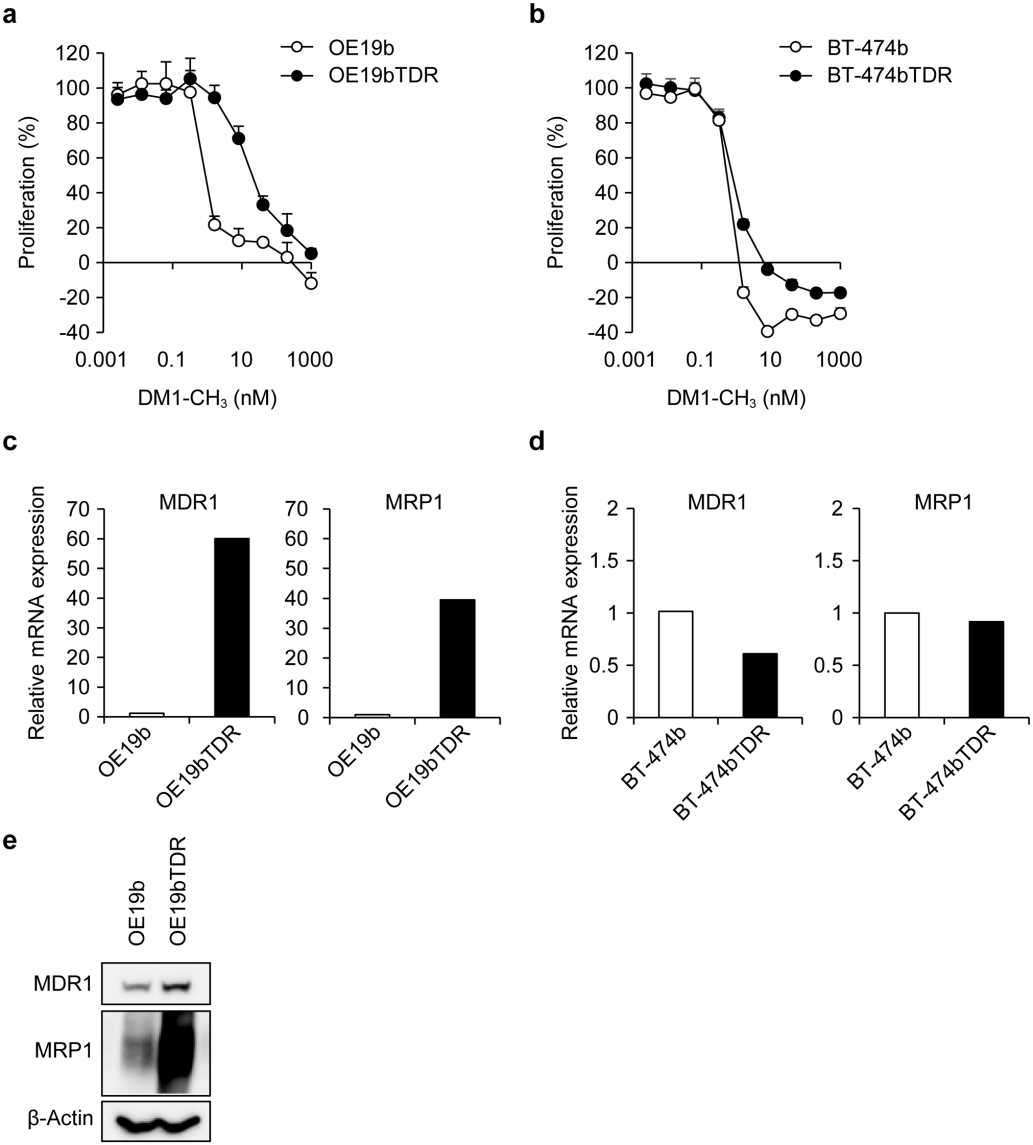
Sensitivity to DM1-CH_3_ and expression of multidrug-resistant proteins in the two T-DM1-resistant cell lines. Cell growth inhibition by DM1-CH_3_ was examined 3 days after treatment in OE19b and OE19bTDR (**a**), and in BT-474b and BT-474bTDR (**b**). Data points are mean + SD (*n* = 3). Relative mRNA expression (mean, *n* = 2) of MDR1 and MRP1 in OE19bTDR cells vs OE19b cells (**c**) and in BT-474bTDR cells vs BT-474b cells (**d**) was measured by quantitative real-time PCR. (**e**) Expression of MDR1 and MRP1 in OE19bTDR vs OE19b cells

Furthermore, we examined whether T-DM1 resistance was due to the resistance of the payload DM1. The sensitivity to DM1-CH_3_, which is the methyl form of DM1, was assessed in the established T-DM1-resistant cell lines because thiol-containing compounds, such as DM1, can form a disulfide-mediated dimer or a mixed disulfide with other thiol-containing substituents in the cell culture medium or intracellularly and are, therefore, not stable [3]. OE19bTDR was also resistant to DM1-CH_3_ (Fig. 2a). The cell cycle arrest after T-DM1 treatment was also examined because DM1-CH_3_ induced the inhibition of tubulin polymerization. Flow cytometry showed T-DM1-induced M phase arrest (G2/M) in the OE19b cells (70% of cells), whereas M phase arrest was suppressed in the OE19bTDR cells (24% of cells) (Online Resource 1a). The gene mutations and expression levels of mitosis-related proteins were not different between resistant and parental cells (data not shown and Online Resource 1b). Sensitivity to DM1-CH_3_ was similar between the BT-474bTDR and the BT-474b cells (Fig. 2b), which is consistent with the results of a previous study [20]. Using real-time polymerase chain reaction (PCR), an increase in relative mRNA expression of MDR1 and MRP1 was observed in the OE19bTDR cells compared with the OE19b cells (Fig. 2c), which was confirmed by western blotting (Fig. 2e); no increase in relative mRNA expression was observed in the BT-474bTDR cells compared with BT-474b cells (Fig. 2d).

### Involvement of MDR1 and MRP1 expression in the T-DM1 resistance of OE19bTDR

**Fig. 3:**
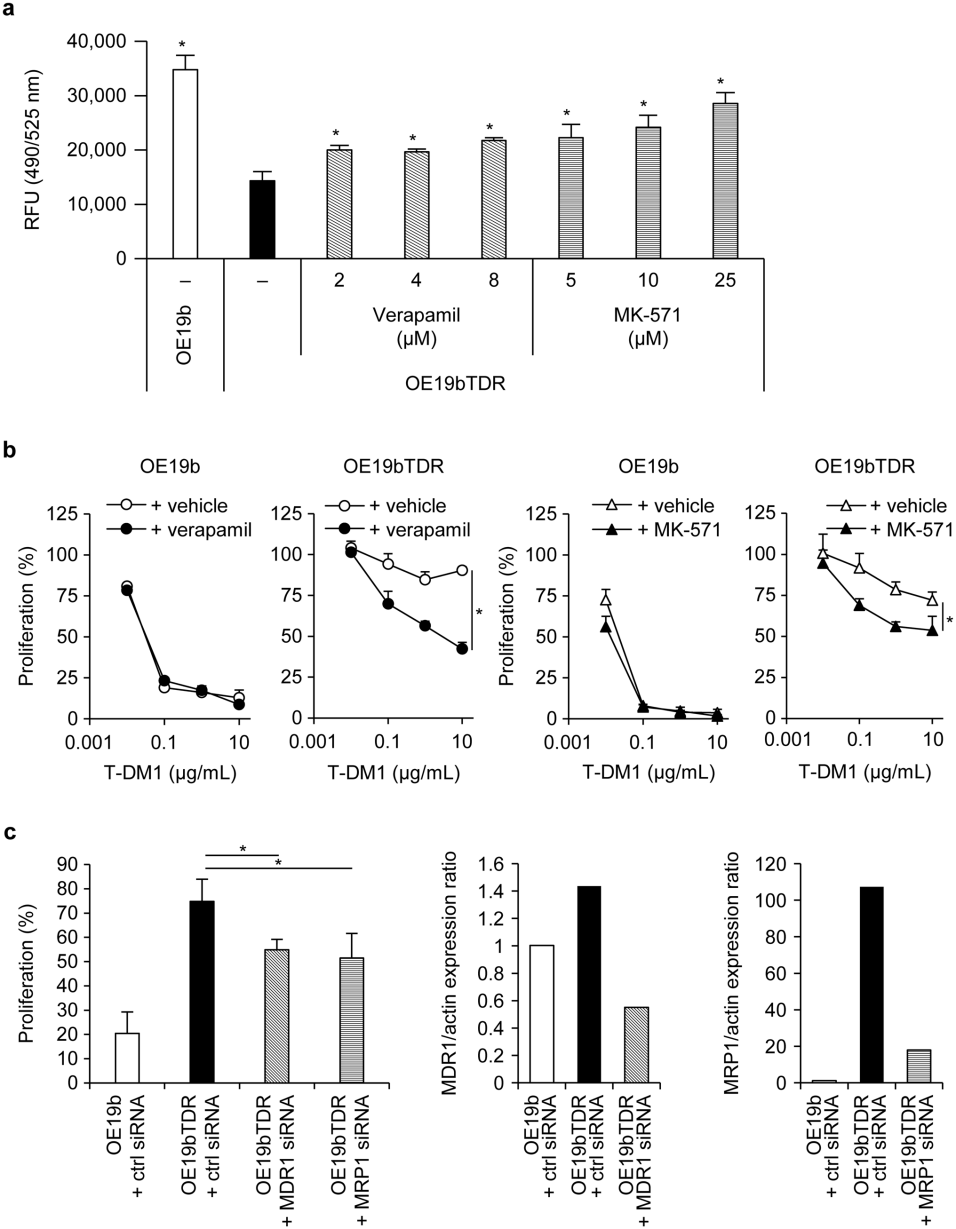
Involvement of MDR1 and MRP1 in the T-DM1 resistance of OE19bTDR cells. **a** Cells were incubated with indicated concentrations of verapamil or MK-571 for 2.5 h and dye efflux by MDRs was measured. Data points are mean + SD (*n* = 3) (*P < 0.05 for all doses; Dunnett test). **b**, **c** Cell growth inhibition by T-DM1 was examined 3 days after treatment in the presence of verapamil (8 μM) or MK-571 (20 μM), **b** or under the condition of *MDR1* or *MRP1* knockdown. **c** Cells were treated with T-DM1 at 0.5 μg/mL, and the proliferation rate was calculated as the ratio of the proliferation of cells transfected with control siRNAs to the proliferation of cells treated with 0 μg/mL of T-DM1. Data points are mean + SD (*n* = 3) (*P < 0.05; Student’s *t* test). MDR1 and MRP1 expression levels were detected by western blotting, and the expression rate compared with that of the parent cell was calculated by a ratio with actin

To assess the activities of the MDR1 and MRP1 expressed on OE19b or OE19bTDR cells, the efflux activity of the fluorescent dye molecule, which was the substrate of MDR1 and MRP1, was determined. The intensity of cellular fluorescence was remarkably lower in the OE19bTDR cells than in the OE19b cells. A significant dose-dependent recovery of cellular fluorescence in the OE19bTDR cells was demonstrated with increasing concentrations of the MDR1 inhibitor verapamil and MRP1 inhibitor MK-571 (Fig. 3a). Consequently, proliferation assays demonstrated significant recovery of T-DM1 sensitivity in the OE19bTDR cells in the presence of verapamil and MK-571 (Fig. 3b). Furthermore, significant recovery of sensitivity to T-DM1 was demonstrated in the OE19bTDR cells after the knockdown of *MDR1* or *MRP1* gene expression by siRNA (Fig. 3c).

### Intracellular uptake of T-DM1

**Fig. 4:**
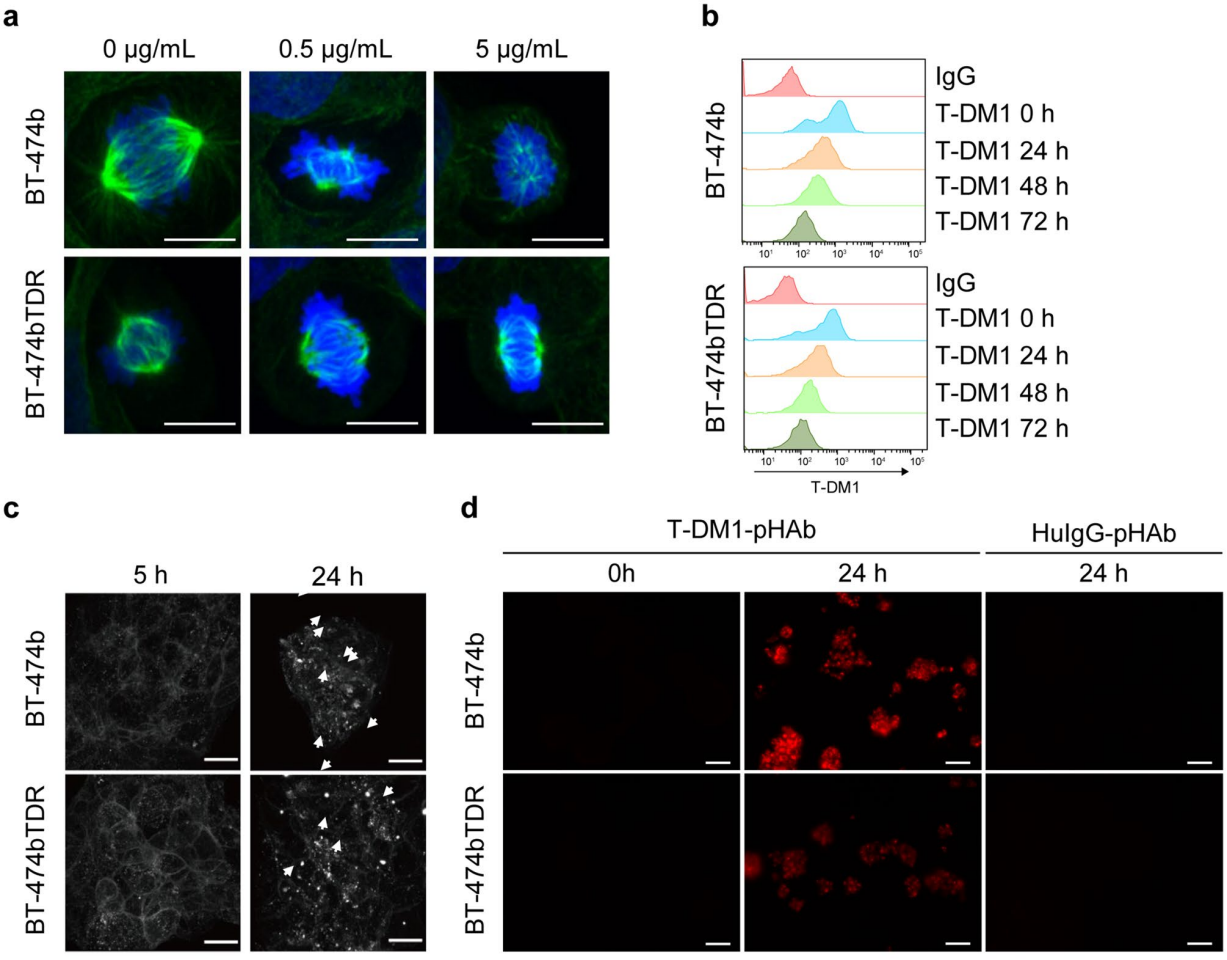
Intracellular uptake of T-DM1 in BT-474b and BT-474bTDR cells. **a** Mitotic spindle formation in T-DM1-treated cells. Cells were treated with T-DM1 for 48 h and tubulin was detected by immunofluorescence staining (green: anti-tubulin antibody; blue: DAPI). White line represents 10 μm. **b** Cell surface T-DM1 was detected by flow cytometry at 0, 24, 48, and 72 h after T-DM1 addition. **c** Alexa-488-labeled T-DM1 was incubated with cells and detected after 24 h incubation using fluorescence microscopy (white arrow: T-DM1). White line represents 20 μm. **d** Lysosomal localization of T-DM1. Cells were incubated with pHAb thiol reactive dye–labeled T-DM1 (T-DM1-pHAb) or HuIgG (HuIgG-pHAb) for 24 h and visualized with fluorescence microscopy. White line represents 100 μm. The figures show a typical dyeing image of multiple experiment results (**a**, **c**, and **d**)

In the BT-474bTDR cells, no increase in expression of MDR1 or MRP1 was observed, and the sensitivity of DM1-CH_3_ was similar to that of the parent cells (Fig. 2b, d). However, staining of tubulin showed that mitotic spindles were not inhibited at concentrations of T-DM1 up to 5 μg/mL in the BT-474bTDR cells compared with concentrations up to 0.5 μg/mL in the BT-474b cells (Fig. 4a). Thus, we examined the process during which receptor-bound T-DM1 was endocytosed into the cell, recruited to lysosome, degraded to DM1 in lysosome, and DM1 released from lysosome to cytosol for inhibiting mitotic spindle formation. The T-DM1 on the cell surface and intracellular uptake of T-DM1 in the BT-474bTDR cells were similar to those in the BT-474b cells (Fig. 4b, c), indicating that endocytosis of receptor-bound T-DM1 was not inhibited in BT-474bTDR cells. For the detection of T-DM1 transported to the lysosome, we used pHAb Amine Reactive Dyes, which fluoresced only in the acidic compartments in cells, for example, in lysosomes (pH 5.0). Lysosomal localization of T-DM1 decreased in the BT-474bTDR cells (Fig. 4d). Despite this decrease, cathepsin activity in lysosome did not decrease in the BT-474bTDR cells (Online Resource 2a). In addition, the expression of SLC46A3 (lysosomal transporter of DM1-based catabolites) was comparable to that of the BT-474b cells (Online Resource 2b). These results indicated that recruiting of T-DM1 to lysosome after endocytosis was inhibited in the BT-474bTDR cells rather than degradation of T-DM1 in lysosome and release of DM1 from lysosome to cytosol.

### Efficacy of the TRAS + PER combination in T-DM1–resistant cell lines

**Fig. 5:**
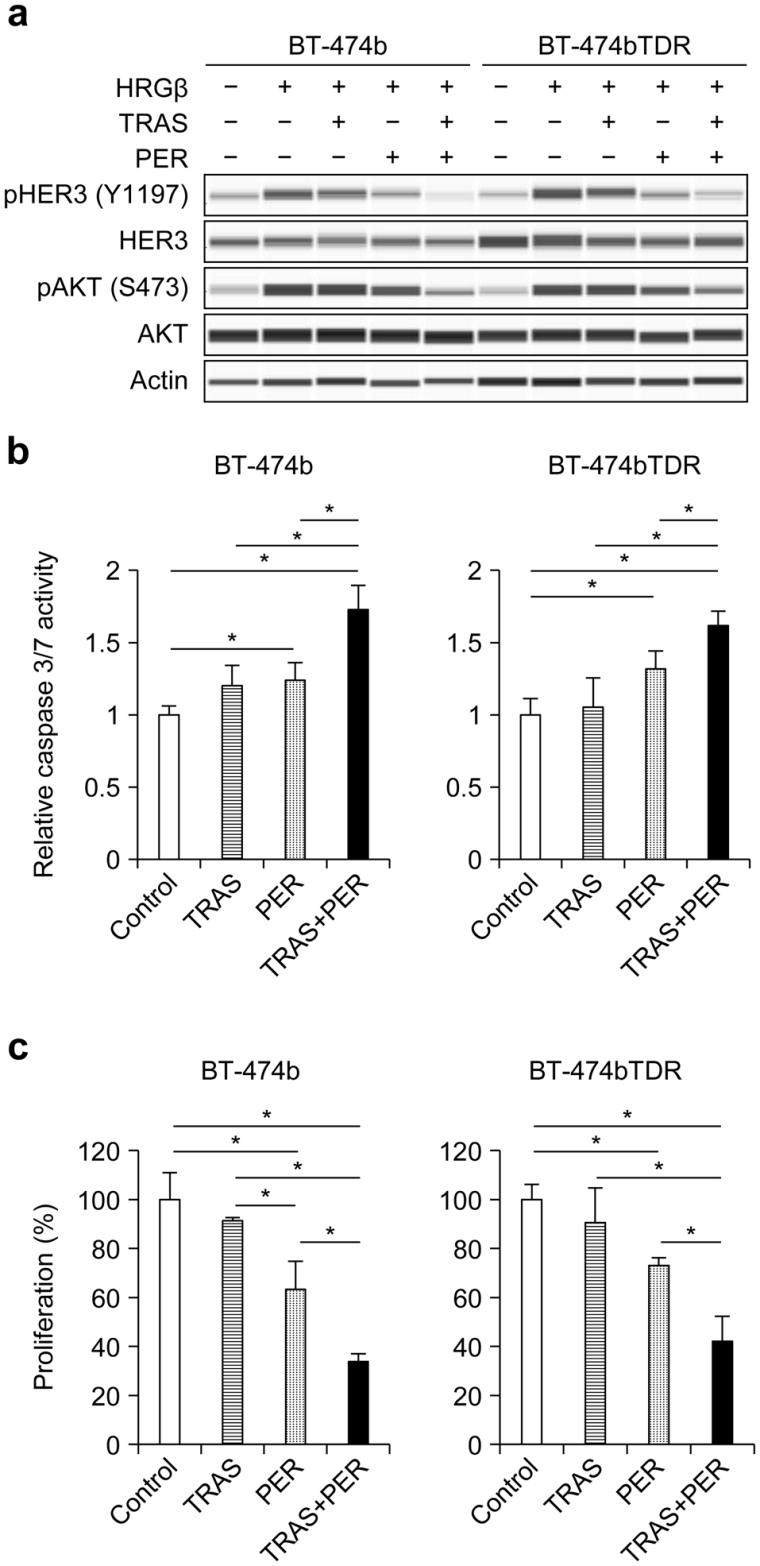
Sensitivity of TRAS + PER in BT-474b and BT-474bTDR cells. **a** Indicated cells were treated with TRAS and PER (40 μg/mL each) for 3.5 h and then incubated with HRGβ (100 ng/mL) for 5 min. Phosphorylation of HER3 and AKT was detected by western blotting. **b** Cells were treated with TRAS and/or PER (40 μg/mL each), and half an hour later, with 40 ng/mL of HRGβ, and then incubated for 24 h. Caspase 3/7 activity was measured using the Caspase-Glo Assay kit. Data plots are mean + SD (*n* = 5/group; *P < 0.05 for all comparisons; Tukey–Kramer’s test). **c** Cells were treated with TRAS and/or PER (40 μg/mL each) for 24 h, then incubated with HRGβ (20 ng/mL), and then treated for 6 days. Cell growth inhibition was examined by Cell Counting Kit-8. Data plots are mean + SD (*n* = 3/group; *P < 0.05 for all comparisons; Tukey–Kramer’s test)

Because HER2 overexpression was maintained in the BT-474bTDR cells (Fig. 1d), the dependence on HER2–HER3 heterodimer signaling was examined. The phosphorylation of HER3 and AKT was similarly inhibited by TRAS + PER treatment in the BT-474b and BT-474bTDR cells (Fig. 5a). Relative caspase 3/7 activity in the BT-474bTDR vs BT-474b cells showed significant induction of apoptosis at 24 h after treatment with PER alone and TRAS + PER compared with controls; apoptosis induction was significantly higher with TRAS + PER than with the individual drugs (Fig. 5b). As a result, a significant reduction in cell proliferation was also observed in the BT-474bTDR vs BT-474b cells at day 7 with PER alone and TRAS + PER compared with controls; inhibition was significantly higher with TRAS + PER than with the individual drugs (Fig. 5c).

**Fig. 6:**
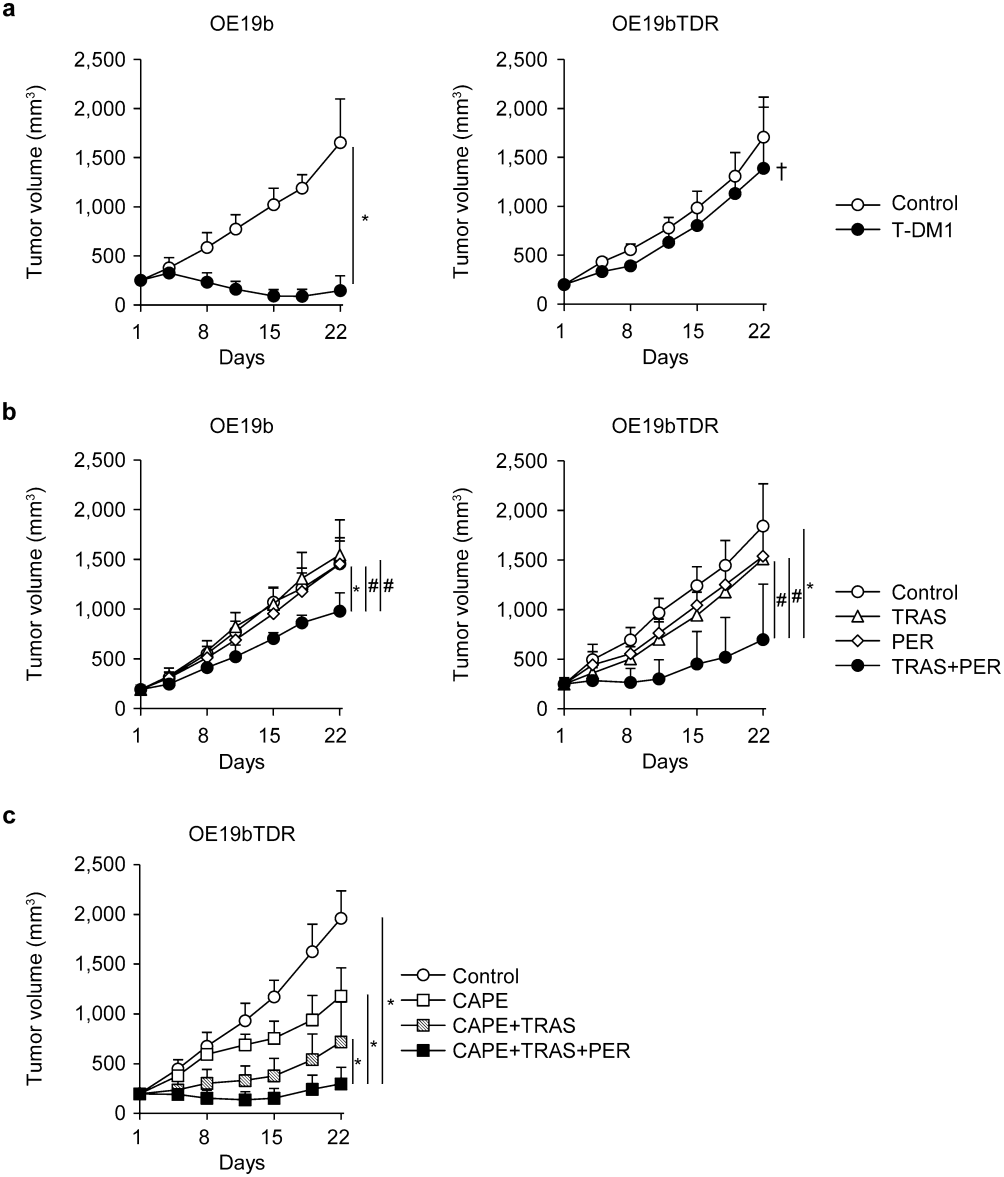
Antitumor activity of the TRAS + PER combination in a T-DM1-resistant cancer xenograft model. **a** Mice bearing OE19b or OE19bTDR tumors were randomly divided into two groups (*n* = 6/group) and treated with saline or T-DM1 (10 mg/kg intravenously once every 3 weeks (*P < 0.05; Wilcoxon test). ^†^One mouse was euthanized, because the tumor volume exceeded the ethical standard of the Institutional Animal Care and Use Committee at Chugai Pharmaceuticals, Co., Ltd. **b** The mice were randomly divided into four groups (*n* = 6/group) and treated with HuIgG, TRAS, PER, or TRAS + PER (80 mg/kg each) intraperitoneally once a week for 3 weeks (*P < 0.05, ^#^*P* < 0.025; Wilcoxon test with Bonferroni correction). **c** OE19bTDR xenografted mice were randomly divided into four groups (*n* = 6/group) and treated with HuIgG, CAPE, CAPE + TRAS, or CAPE + TRAS + PER. TRAS, PER, or HuIgG was administered intraperitoneally at 40 mg/kg once a week for 3 weeks; CAPE (359 mg/kg) or CAPE vehicle was administered perorally for initial 14 days (*P < 0.05; Wilcoxon test)

The OE19bTDR xenografted tumors were also resistant to T-DM1 treatment compared with the OE19b tumors (Fig. 6a). Because of the lower sensitivity of OE19b to TRAS or PER in the xenograft model, mice with OE19b and OE19bTDR tumors were treated with a higher dose. Tumor growth was significantly suppressed with the TRAS + PER combination compared with TRAS and PER alone (tumor growth inhibition rates on day 22 were 19% with TRAS, 18% with PER, and 58% with TRAS + PER in the OE19bTDR xenograft model) (Fig. 6b).

In the combination treatment with CAPE, a chemotherapeutic agent used as the third-line drug in combination with anti-HER2 therapy, the OE19bTDR tumors were sensitive to CAPE and CAPE + TRAS treatment. Furthermore, the addition of PER to the CAPE + TRAS combination led to a significant decrease in tumor volume in the OE19bTDR xenograft model (Fig. 6c).

## Discussion

The molecular mechanisms that drive clinical resistance to T-DM1, especially in HER2-positive tumors, are not well understood. In the OE19bTDR cells, suppression of MDR1 or MRP1 by each inhibitor or siRNA partially restored the cytotoxic activities of T-DM1, and result from RNA sequence analysis showed no increase in other ABC transporters, including ABCB2 or ABCG2 (data not shown), indicating that expression of MDRs was predominantly associated with T-DM1 resistance. Previous studies have also reported on the overexpression of ABC transporters as resistant mechanisms of T-DM1. Le Joncour et al. reported that upregulation of ABCC1/MRP1 and ABCC2/MRP2 caused T-DM1 resistance in the OE19 cell line and ABCC2 and ABCG2/breast cancer resistance protein (BCRP) in the NCI-N87 cell line [22]. Sauveur et al. reported alterations to cell adhesion molecules using the OE19 cell line [30]. Loganzo et al. reported that overexpression of ABCC1-induced T-DM1 resistance in 361 cell lines [18], and Li et al. reported that ABCB1 overexpression and HER2 downregulation were causes of T-DM1 resistance in KPL-4 cells [20]. Thus, overexpression of ABC transporters may inherently contribute to T-DM1 resistance and could be one of the representative characters of T-DM1 resistance.

In BT-474bTDR cells, T-DM1 uptake from the cell membrane to cytosol followed by trafficking to endosome was not impaired. However, the amount of T-DM1 in the lysosome was decreased, and neither a loss of lysosome enzyme activity nor a loss of the lysosomal transporter SLC46A3 was observed. These results suggested that the mechanism of T-DM1 resistance in BT-474bTDR could be decreased localization of T-DM1 into the lysosomes without loss of lysosomal activity. Rios-Luci et al. reported that impaired lysosomal proteolytic activity is one of the T-DM1 resistant mechanisms [24], whereas Li G et al. reported loss of lysosomal transporter SLC46A3 and PTEN deficiency in BT-474M1 cells [20]. Kinneer et al. reported SLC46A3 loss in SK-BR-3 cells [31], and Wang et al. reported lysosome acidification decrease (vacuolar H + -ATPase decrease) in NCI-N87 cells [25]. Thus, although several mechanisms could exist, deficiency of DM1 release from lysosome may be another representative resistance mechanism, and BT-474bTDR would have an equivalent resistance mechanism to this class.

The trafficking abnormality of T-DM1 to lysosomes due to endocytosis by caveolin-1 is implicated in T-DM1 resistance [26]. Although caveolin-1 is responsible for the internalization of HER2 molecule and affects TRAS efficacy [32], its overexpression was not observed in BT-474bTDR cells (data not shown). Further studies are necessary to elucidate the mechanism of resistance in BT-474bTDR cells. Despite the trafficking abnormality of membrane-bound molecules, HER2 expression level was maintained and HER2 signal inhibition was effective in BT-474bTDR. Therefore, in cancers that maintain HER2 overexpression after T-DM1 resistance similar to that in the BT-474bTDR cells, signal inhibitors such as TRAS and PER, other than antibody–drug conjugates, might be a reasonable treatment.

It is recognized that among the different known MOAs, the combination of TRAS + PER induces inhibition of both ligand-dependent and ligand-independent HER2–HER3 signaling [5, 33]. In this study, the combination of TRAS + PER induced the inhibition of phosphorylation of downstream factors involved in HER2–HER3 heterodimer signaling, and apoptosis induction and reduction in cell proliferation were significantly higher with the TRAS + PER combination than with the individual drugs in the BT-474bTDR and BT-474b cells. Furthermore, tumor growth was significantly suppressed with the TRAS + PER combination in the OE19bTDR xenograft model compared with either TRAS or PER alone.

In clinical practice, TRAS or TRAS + PER is used in combination with other chemotherapy drugs [10–12]. In case the T-DM1-resistant tumor has sensitivity to chemotherapeutic drugs, it is likely that PER and TRAS in combination with chemotherapy will be more effective. Therefore, we explored the antitumor activity of CAPE, TRAS, and PER. We observed that the OE19bTDR xenografted tumors were sensitive to CAPE and CAPE + TRAS and that the triple combination of CAPE + TRAS + PER showed significant antitumor effect compared with CAPE + TRAS in the xenograft model, although PER alone did not show antitumor activity (Fig. 6b, c). This suggests that TRAS + PER in combination with chemotherapy such as CAPE may be useful for T-DM1-resistant cancers caused by overexpression of MDR1 and MRP1 by proper selection of chemotherapy drugs.

Although our present study has some limitations, because the T-DM1-resistant cells in this study were established from cells naïve for both TRAS and PER despite the clinical use of T-DM1 after the regimen including TRAS and PER, our results showed the potential of TRAS + PER + chemotherapy as third-line combination therapy after T-DM1 resistance if cancer cells remain dependent on HER2.

The clinical application of our findings is being evaluated in an ongoing multicenter, randomized, open-label, phase 3 study (PRECIOUS trial). This clinical study aims to demonstrate the usefulness of PER re-administration after resistance to T-DM1 in HER2-positive locally advanced/metastatic breast cancer patients with a history of PER administration. This study is based on the premise that if HER2–HER3 signaling, which was suppressed by previously used PER-containing regimens, is restored during anti-HER2 therapy without PER, such as T-DM1 therapy before re-administration of a PER-containing regimen, PER re-administration might potentially re-suppress HER2–HER3 signaling [34]. If the efficacy of PER re-administration is demonstrated in the PRECIOUS trial, then PER re-administration may become the standard third- and subsequent-line therapy for HER2-positive locally advanced/metastatic breast cancer.

## Conclusion

The results of this study suggest that the combination of TRAS + PER may be effective in T-DM1-resistant cancer where HER2 overexpression is maintained.

## Electronic supplementary material

Below is the link to the electronic supplementary material.

Supplementary file1 (PDF 212 kb)

## Abbreviations

ABC: ATP-binding cassette
ABCB1: ATP-binding cassette subfamily B member 1
ABCC1: ATP-binding cassette subfamily C member 1
ADCC: Antibody-dependent cellular cytotoxicity
ATP: Adenosine triphosphate
BCRP: Breast cancer resistance protein
CAPE: Capecitabine
CDC20: Cell-division cycle protein 20
CDK1: Cyclin-dependent kinase 1
EGFR: Epidermal growth factor receptor
HER2: Human epidermal growth factor receptor 2
HRGβ: Heregulin β
HuIgG: Human immunoglobulin G
IC_50_: Half maximal inhibitory concentration
IHC: Immunohistochemistry
MDR1: Multidrug resistance protein 1
MOA: Mechanism of action
mRNA: Messenger RNA
MRP1: Multidrug resistance-associated protein 1
MUC4: Mucin 4
PBS: Phosphate buffered saline
PCR: Polymerase chain reaction
PER: Pertuzumab
PTEN: Phosphatase and tensin homfolog
RT: Room temperature
siRNA: Small interfering RNA
SLC: Solute carrier
T-DM1: Trastuzumab emtansine
TRAS: Trastuzumab
