# Supplementary material for: Combination efficacy of pertuzumab and trastuzumab for trastuzumab emtansine-resistant cells exhibiting attenuated lysosomal trafficking or efflux pumps upregulation
Source: Cancer Chemother Pharmacol. 2020 Sep 30;86(5):641–54. doi: 10.1007/s00280-020-04138-5 (PMC7561595; doi:10.1007/s00280-020-04138-5)
Supplement: Supplementary file 1 — Supplementary file1 (PDF 212 kb) [file 280_2020_4138_MOESM1_ESM.pdf]

Online Resource 1

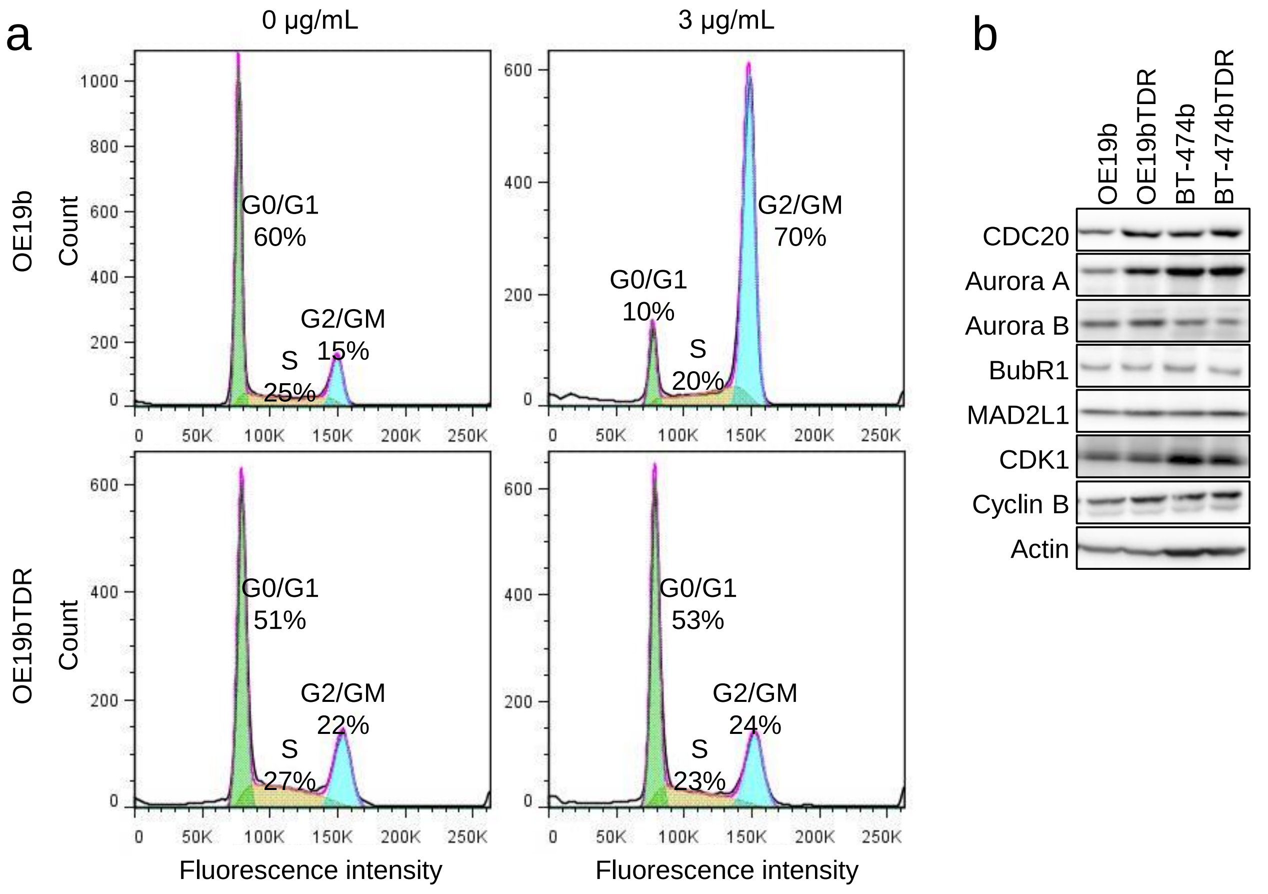

Online Resource 1 (**a**) Cell cycle in OE19b and OE19bTDR cells after T-DM1 treatment. OE19b and OE19bTDR cells seeded in 25 cm^2 flasks at 5 x 10^5 cells and precultured for 24 h were treated with 3 µg/mL of T-DM1 and incubated for 24 h. Cells along with the culture medium were collected and prepared for cell cycle analysis using a BD Cycletest Plus DNA Reagent Kit. The samples were analyzed on a flow cytometer and FlowJo v7.6.5. (**b**) Expression levels of mitosis-related proteins were detected by western blotting.

CDC20, cell-division cycle protein 20; CDK, cyclin-dependent kinase;
T-DM1, trastuzumab emtansine.

Online Resource 2

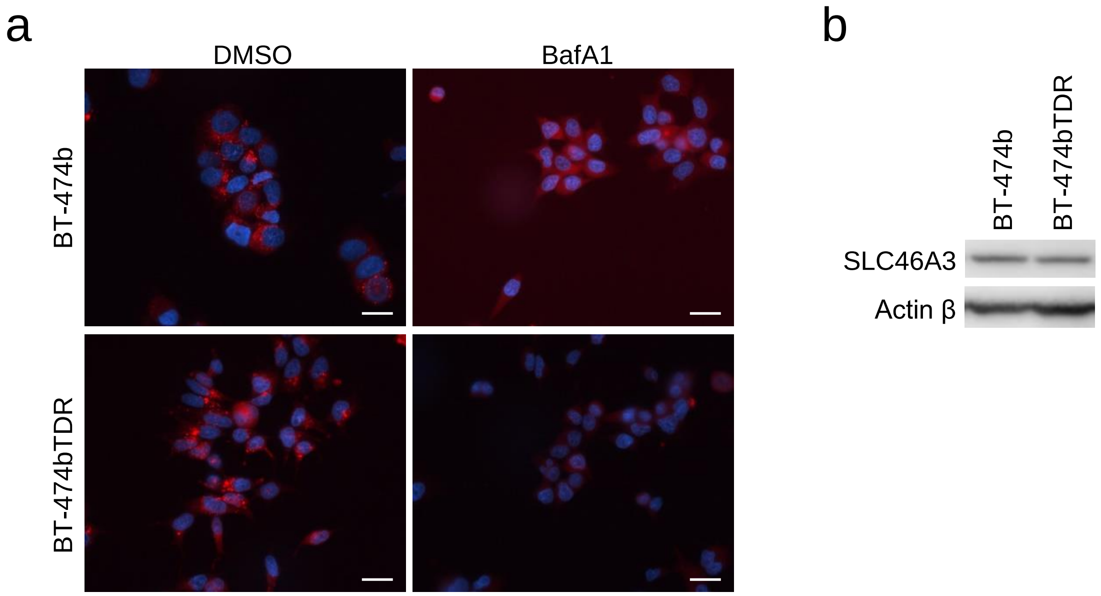

Online Resource 2 Lysosomal activity in BT-474b and BT-474bTDR cells.
(**a**) BT-474b and BT-474bTDR cells were seeded on 8-well chamber slides at 3×10^4 cells/well and precultured for 24 h. The cells were then treated with DMSO or 1 μM BafA1 (cathepsin inhibitor) and incubated for 2 h. After changing the medium, activated lysosomes were detected by a Magic Red Cathepsin Assay Kit, and fluorescence microscopy was performed to visualize the cells. − represents 20 μm. The figure shows a typical dyeing image of multiple study results. (**b**) SLC46A3 expression was detected by western blotting in the BT-474b and BT-474bTDR cells.
BafA1, bafilomycin A1; DMSO, dimethyl sulfoxide; SLC46A3, solute carrier family 46 member 3.
